# Bi-exponential 3D-T1ρ mapping of whole brain at 3 T

**DOI:** 10.1038/s41598-018-19452-5

**Published:** 2018-01-19

**Authors:** Rajiv G. Menon, Azadeh Sharafi, Johannes Windschuh, Ravinder R. Regatte

**Affiliations:** Bernard and Irene Schwartz Center for Biomedical Imaging, New York University School of Medicine, New York, NY USA

## Abstract

Detection of multiple relaxation pools using MRI is useful in a number of neuro-pathologies including multiple sclerosis (MS), Alzheimer’s, and stroke. In this study we evaluate the feasibility of using T1ρ imaging for the detection of bi-exponential decays in the human brain. A prospective T1ρ imaging study was performed on model relaxation phantoms (eggs) and 7 healthy volunteers. The data was fitted using a single pool and a 2-pool model to estimate mono- and bi-exponential T1ρ maps, respectively. Bi-exponential decays were identified in the gray matter (GM) and white matter (WM) of the brain with 40.5% of GM, and 65.1% of WM pixels showing two T1ρ relaxation pools (significance level P < 0.05). Detection of T1ρ based bi-exponential decays in the brain provides complimentary information to T_2_ based contrast regarding the *in vivo* micro-environment in the brain.

## Introduction

Detection and characterization of multiple components of relaxation (MCR) in the brain is useful, particularly in the assessment of myelin content, in understanding normal brain development^1^ and in neuro-pathologies. A number of MCR based studies using T_2_ relaxation have demonstrated its utility in assessing white matter pathology including multiple sclerosis (MS)^2^, Alzheimer’s^3^, and stroke^4^. MRI techniques such as multi-echo T_2_^2^, 3D gradient and spin echo (GRASE)^5^, and multi-component driven equilibrium single pulse observation of T_1_ and T_2_ (mcDESPOT)^6^ have been developed to use T_2_ to assess myelin.

T1ρ refers to the spin lattice relaxation time constant in the rotating magnetic field, and measures the transverse magnetization decay in the presence of a spin-lock radiofrequency (RF) field^7^. While the processes that give rise to T_1_ and T_2_ contrast related to molecular rotation are in the Larmor time-scale, with frequencies on the order of megahertz, the T1ρ contrast mechanism is sensitive to a lower frequency (kilohertz) range, and picks up signal from lower energy interactions related to chemical exchange between extracellular water and complex macromolecules^8^. T1ρ imaging has been extensively used to quantify proteoglycan content in articular cartilage^9–11^, and is a promising approach to characterize cartilage degradation. In the brain, most studies have used mono-exponential T1ρ imaging to evaluate a number of neuro-pathologies^12,13^. While bi-exponential decays using T1ρ have been detected in cartilage^14,15^ and muscle^16^, we did not find published reports of T1ρ bi-exponential decay in the brain.

In this paper, we demonstrate the feasibility of T1ρ imaging to estimate bi-exponential relaxation mapping of whole brain. By using phantom experiments using avian eggs, and healthy volunteers we show that bi-exponential decays can be detected in the human brain *in vivo*.

## Results

### Model Phantoms

**Figure 1:**
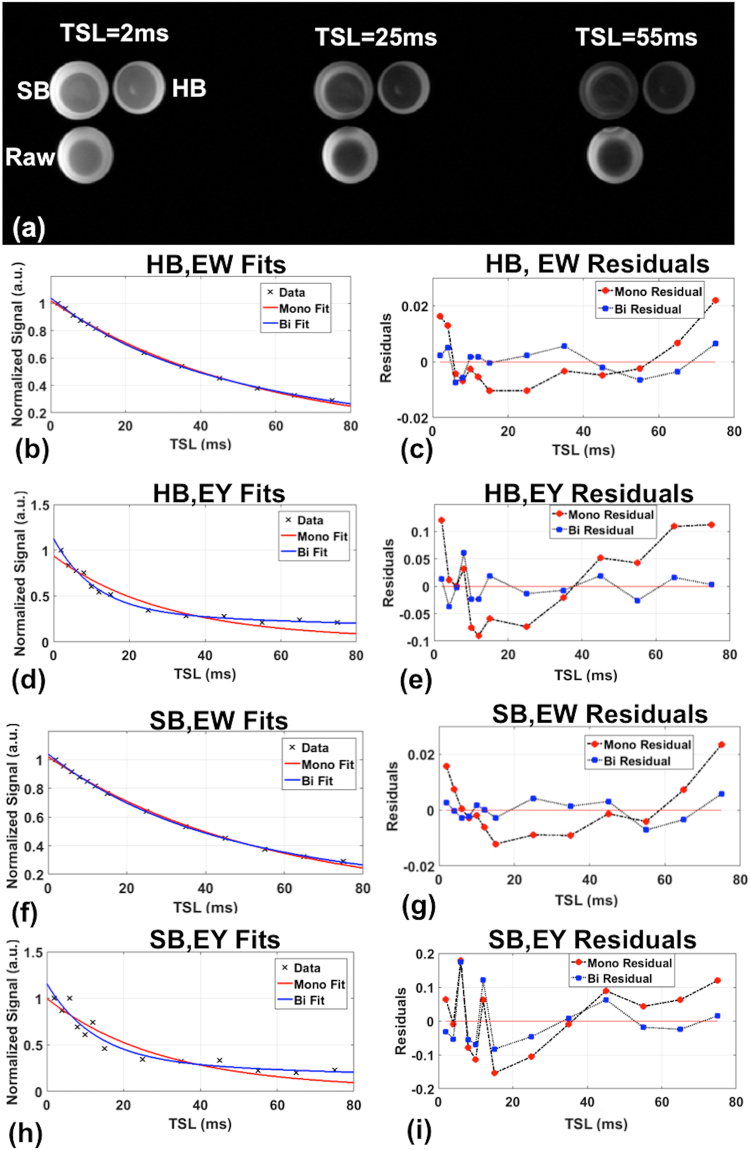
Sample egg images of HB, SB and raw egg at three representative TSL values (**a**). Signal decays with mono- and bi-exponential fits in the hard-boiled egg white (**b**), yolk (**d**), their residuals (**c**,**e**) and soft-boiled egg white (**f**), yolk (**h**) and their residuals (**g**,**i**).

Figure 1 and Table 1 show results from the egg experiments. Figure 1(a) shows representative images at selected TSL values of the hard boiled (HB), soft-boiled (SB) and raw eggs. Bi-exponential decays (short relaxation time, T1ρs and long relaxation time, T1ρl) were detected in both the egg white and yolk of the HB, SB and raw eggs. For the hard-boiled egg whites, although the mono-exponential and bi-exponential fits seem good fits to the data (Fig. 1(b)), the residuals (Fig. 1(c)) clearly demonstrate that the bi-exponential is a better fit. For the hard-boiled egg yolk, bi-exponential decays are clearly better fits to the data (Fig. 1(d)) and with lower residuals (Fig. 1(e)). Results in soft-boiled egg-white and egg yolks follow a similar trend as the hard-boiled counterparts (Fig. 1 (f–i)). In the raw egg, the mono-exponential T1ρ relaxation time for the yolk had similar values to HB, and SB cases, but the egg white showed significantly longer mono-exponential T1ρ relaxation times compared to SB or HB egg-white (Table 1). The egg-white T_1ρs_ and T_1ρl_ reduced 40.6% and 21.2% from soft-boiled to hard-boiled respectively, while the yolk underwent a reduction of 15.2% and 11.6% for the state change from soft-boiled to hard-boiled. Relaxation times, fractions and goodness of fits (adjusted R^2^) for the egg-white and yolk for the HB, SB and raw eggs are reported in Table 1.

**Table 1:** Egg experiments fitting results (N = 2) and volunteer human brain fitting results (N = 7). EW-eggwhite, EY- eggyolk, HB-hard-boiled, SB-soft-boiled, GM-Gray Matter, WM-white matter, GM+WM- gray and white matter.

ROI		Mono-Exponential	Bi-exponential			
		T1ρm (ms)	T1ρs (ms)	T1ρl(ms)	As(%)	Al(%)
**EGGS**						
EW HB	Mean ± SD	50.66 ± 9.93	14.87 ± 11.68	82.80 ± 41.50	39.47 ± 20.96	60.53 ± 20.96
	R^2^	0.99069	0.99187	0.99187	—	—
EW SB	Mean ± SD	61.29 ± 15.23	25.05 ± 15.73	105.17 ± 46.47	39.84 ± 26.26	60.16 ± 26.26
	R^2^	0.91661	0.97811	0.97811	—	—
EWRaw	Mean ± SD	140.11±14.68	6.14 ± 5.08	230.72 ± 55.94	21.34 ± 16.88	78.62 ± 16.88
	R^2^	0.8883	0.8925	0.8925	—	—
EY HB	Mean ± SD	31.22 ± 3.32	13.46 ± 2.52	140.53 ± 30.75	71.88 ± 5.43	28.12 ± 5.43
	R^2^	0.94932	0.97745	0.97745	—	—
EY SB	Mean ± SD	36.00 ± 8.06	15.88 ± 4.74	158.96 ± 10.93	70.75 ± 9.19	29.25 ± 9.19
	R^2^	0.77214	0.97847	0.97847	—	—
EY Raw	Mean ± SD	31.14 ± 8.86	8.3 ± 0.54	299.86 ± 11.49	69.38 ± 6.65	30.62 ± 6.65
	R^2^	0.8994	0.9008	0.9008	—	—
**IN- VIVO**						
GM	Mean ± SD	72.25 ± 3.39	10.41 ± 6.89	82.87 ± 17.60	14.74 ± 4.96	85.26 ± 4.96
	95% CI	69.61–75.88	9.62–15.46	66.59–99.14	10.12–19.32	80.68–89.88
	R^2^	0.9799	0.9907	0.9907	—	—
WM	Mean ± SD	67.43 ± 2.33	10.09 ± 6.06	78.26 ± 13.69	14.39 ± 4.91	85.61 ± 4.91
	95% CI	65.26–69.57	9.00–14.98	65.59–90.92	9.85–18.94	81.06–90.15
	R^2^	0.9918	0.9972	0.9972	—	—
GM + WM	Mean ± SD	69.80 ± 2.62	10.11 ± 6.40	79.96 ± 15.52	14.55 ± 4.83	85.45 ± 4.83
	95% CI	67.38–72.22	9.43–15.04	65.61–94.31	10.08–19.02	80.98–89.92
	R^2^	0.9857	0.9939	0.9939	—	—

### ***In Vivo*** Results

**Figure 2:**
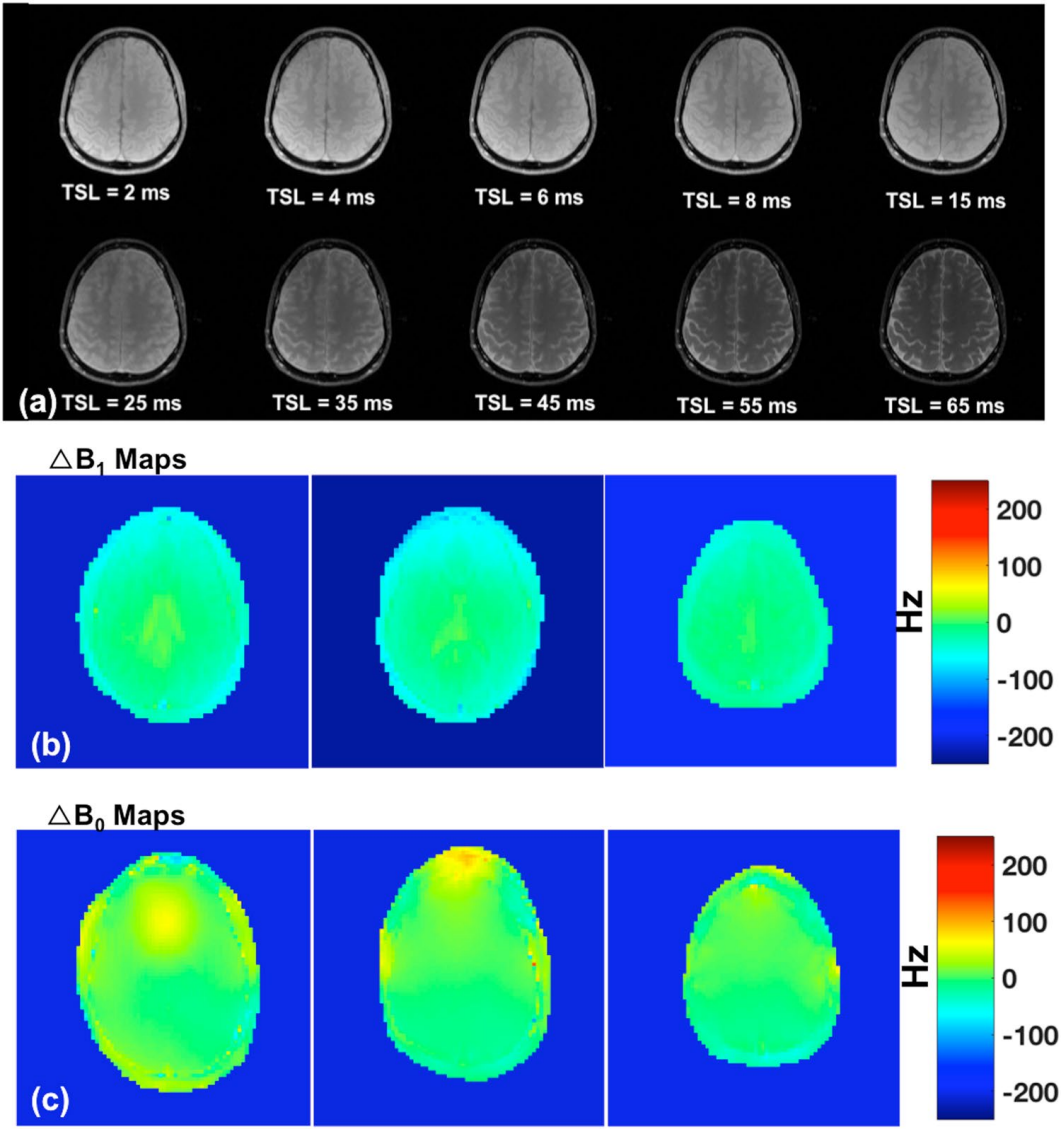
(**a**) Representative T1ρ weighted images of the brain acquired at ten TSL intervals (**b**) ΔB_1_ field map and (**c**) ΔB_0_ field maps.

Figure 2(a) shows T1ρ images at different TSL intervals varying from 2 to 65 ms. Figure 2(b) shows the B_1_ field map changes (ΔB_1_) and Fig. 2(c) shows B_0_ field map changes (ΔB_0_) respectively. The minimum-maximum variation in B_1_ across the imaging sample was between −234.44 to 18.33 Hz, and the minimum to maximum variation in B_0_ was between −206.9 Hz to 182.8 Hz. In our T1ρ experiment we used spin-lock frequency of 500 Hz (higher than ΔB_0_ and ΔB_1_) with 180° refocusing pulse and paired self-compensated spin-lock pulses for T1ρ preparation to minimize imperfections in RF pulses.

**Figure 3:**
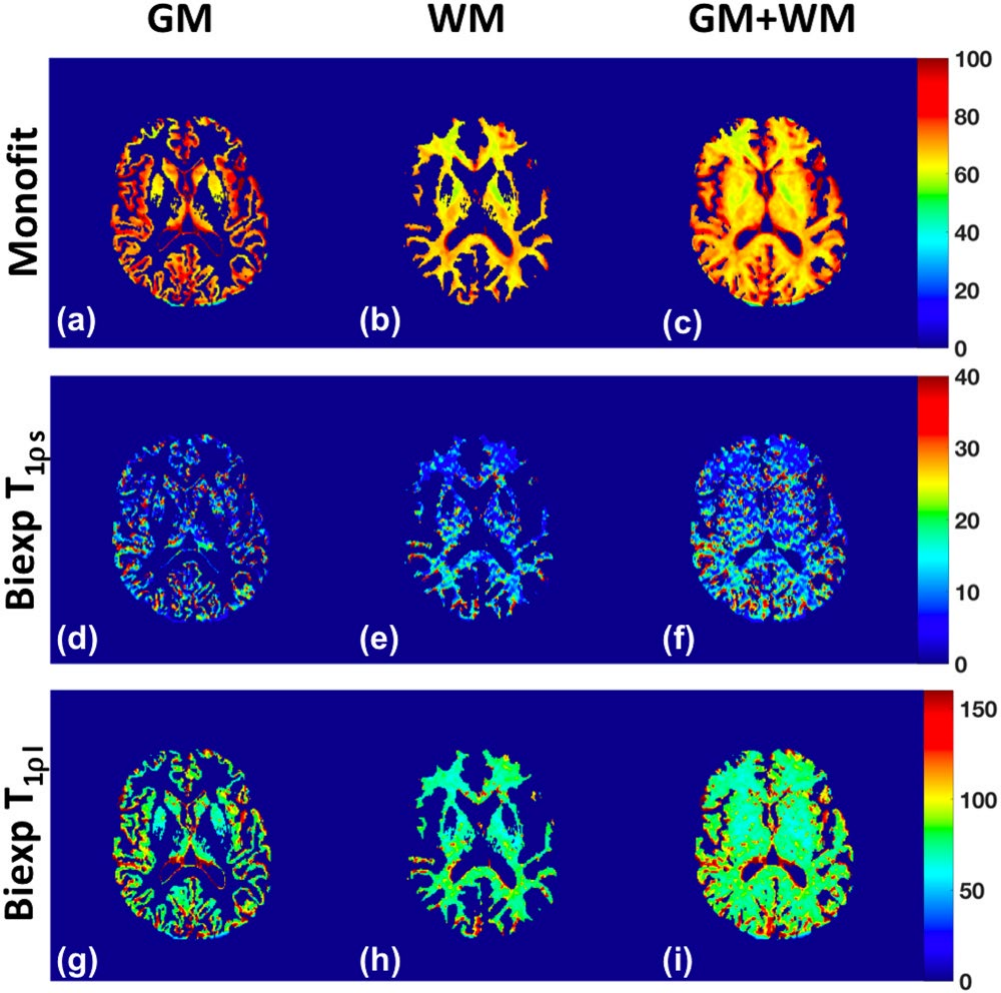
T1ρ maps of a representative brain slice using mono-exponential fit (row 1) and bi-exponential fits (T_1ρs_ component (row 2), T_1ρl_ component (row 3)). The T1ρ map of the gray matter is shown in figures (**a**), (**d**), and (**g**), the white matter in (**b**), (**e**), and (**h**), and gray and white matter shown together in figures (**c**), (**f**), and (**h**).

Figure 3 shows computed T1ρ maps for GM, WM and GM+WM using mono-exponential decay (Fig. 3 (a–c)), the short relaxation component, T_1ρs_ (Fig. 3(d–f)), and the long relaxation component, T_1ρl_ (Fig. 3(g–h)). Table 1 summarizes the mean calculated values across the 7 volunteers. The bi-exponential fit shows that in the GM, there is a fast relaxing (T_1ρs_ = 10.41 ± 6.89 ms) minor component (A_s_ = 14.74 ± 4.96%), while in the WM too, there is a fast relaxing (T_1ρs_ = 10.09 ± 6.06 ms) minor component (A_s_ = 14.39 ± 4.91%). The dominant component in the GM (T_1ρl_ = 82.87 ± 17.60 ms, A_l_ = 85.26 ± 4.96%) and WM (T_1ρl_ = 78.26 ± 13.69 ms, A_l_ = 85.61 ± 4.91%) is the slow relaxing component. The results for the GM+WM fall between the individual GM and WM results. The goodness of fit measure for all cases is above 0.97, indicating an excellent fit to the data (Table 1). The results from the mono-exponential (T_1ρm_) fitting fall between the short and long component values of the bi-exponential fits for the GM (72.25 ± 3.39 ms) and the WM (67.43 ± 2.33 ms). The 95% confidence intervals (CI) of the T1ρ estimates are shown in Table 1. Bi-exponential decays were identified in 40.5% of GM, and 65.1% of WM pixels (with a significance level P < 0.05).

**Figure 4:**
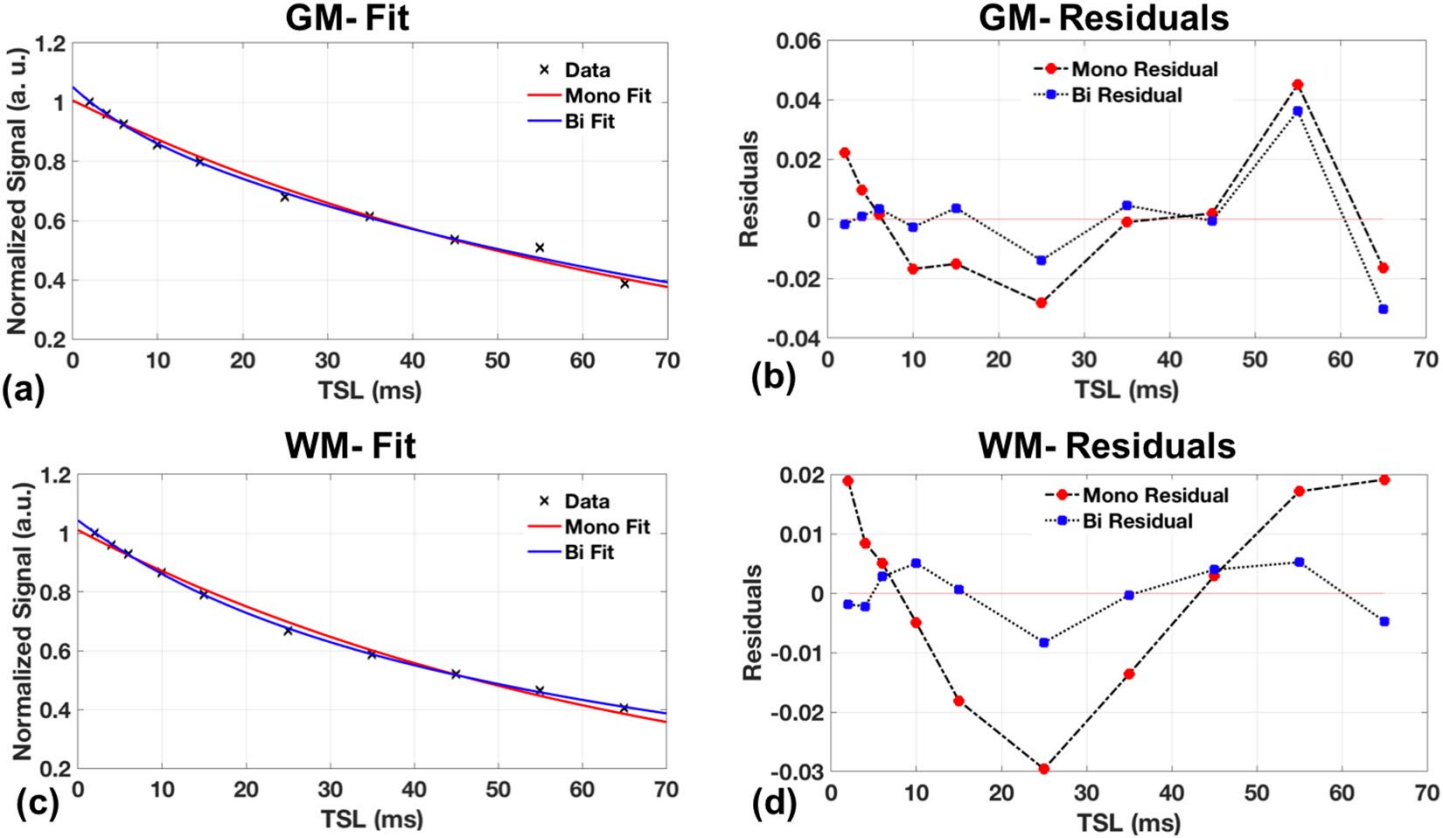
Signal decays from representative voxels with mono- and bi-exponential fits in the gray-matter of the brain (**a**), and the white matter of the brain (**c**) with the corresponding residuals (**b**,**d**). The WM fits and residuals clearly indicate bi-exponentials as a better fit to the data, compared to the GM.

Figure 4 shows the comparison of the mono- and the bi-exponential decays from representative areas of the GM and WM. In the GM the fits seem similar, but the residuals indicate that the bi-exponential fit may be a better fit. In the WM, it is clear from the residuals that the bi-exponential are a better fit to the data. This suggests the delineation of two water pools in the WM, one possibility being the myelin bound water, and the other intra- and extra-cellular water.

**Figure 5:**
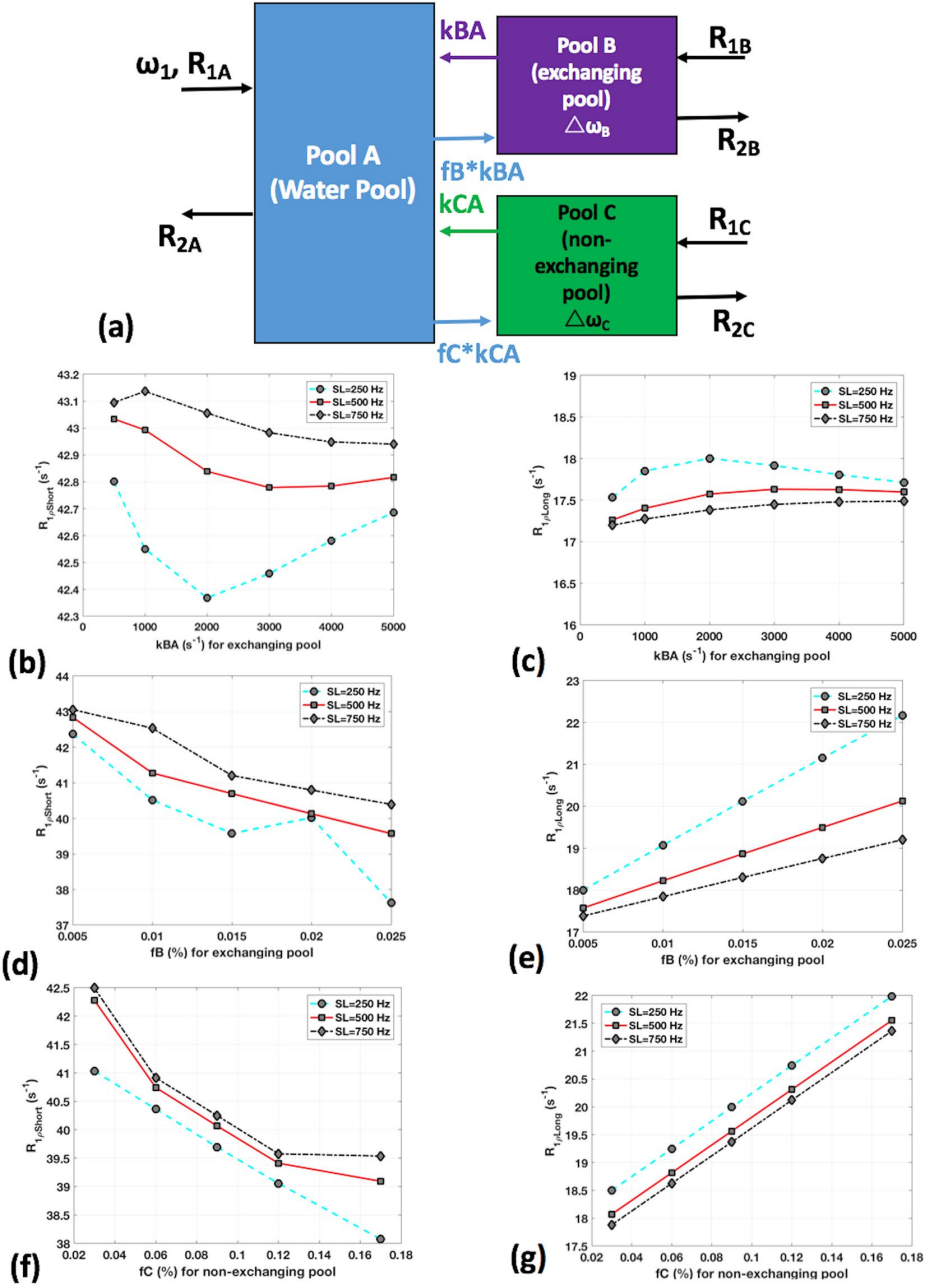
(**a**) shows the Bloch-McConnell simulation with a 3 pool model (water pool, exchanging pool, non-exchanging pool). R1ρs (**b**) and R1ρl (**c**) variation at different spin-lock powers with exchange rate variation in the exchanging pool, *k*_BA_, R1ρs (**d**) and R1ρl (**e**) with solute concentration variation in the exchanging pool, *f*_B_ and, R1ρs (**f**) and R1ρl (**g**) with solute concentration variation in the non-exchanging pool, *f*_C._

Figure 5(b–g) shows the results of Bloch-McConnell simulation. The variation of exchange rate, *k*_BA_ with the rate of T1ρs relaxation (R1ρs = 1/T1ρs) and rate of T1ρl relaxation (R1ρl = 1/T1ρl) are shown in Fig. 5(b) and Fig. 5(c), respectively. Figure 5(d) and (e) shows the short and long R1ρ and its variation with the concentration of the exchanging pool. Figure 5(f) and (g) shows the short and long R1ρ estimates with the variation in concentration of non-exchanging pool. All cases shows the fitted numerical data for spin-lock frequencies of 250, 500 and 750 Hz. The behavior of *k*_BA_, *f*_B_ and *f*_C_ varies with the short and long R1ρ components. In the fast exchanging pool, *k*_BA_, the R1ρs varies significantly based on spin-lock power, while for the long component, R1ρl, there is little effect of exchange rates or spin lock power. Increase in the concentration of the exchanging pool, *f*_*B*_ and the concentration of the non-exchanging pool, *f*_C_ causes a linear reduction in the R1ρs component, and a linear increase in the R1ρl component.

## Discussion

In this study, we demonstrate the feasibility of measuring bi-exponential decays in the whole brain using T1ρ imaging. We tested the T1ρ MCR technique on chicken eggs, recently shown to consist of at least two relaxation components^17^. Additionally, the 3D T1ρ sequence allows for whole brain imaging near isotropic resolution in ~19 minutes and may be potentially useful in a clinical setting. This is the first study to our knowledge that utilizes T1ρ imaging to identify bi-exponential decays in the human brain.

In our study, T_1ρs_ in the brain was in the range of 3.52 to 17.30 ms with a minor fast relaxing component, and a T_1ρl_ component ranging from 65.27 to 100.47 ms with a dominant slow relaxing component. The range of TSL values used in our study was from 2–65ms for *in vivo* studies. Appreciable improvements in T1ρ quantification with longer TSL values in egg experiments were not noted. For *in vivo* studies, additional SAR limitations come into play for longer spin lock durations.

The results from the brain T1ρ bi-exponential fitting reveal a minor fast relaxing component and dominant slow relaxing water compartment, with similar relaxation fractions in the GM and WM of the brain. A previously reported study involving rats did not observe bi-exponential decays in the rat brains^18^, which may likely be attributed to experimental limitations. Mono-exponential T1ρ studies by Gonyea *et al*.^19^ reported a 2% difference between GM and WM in controls, while T1ρ studies by Borthakur *et al*.^12^ at 1.5 T reported a 5% difference between GM and WM. Mangia *et al*.^20^ showed a much wider difference of 20% between GM and WM in their adiabatic T1ρ studies. In our studies the T_1ρl_ component showed a difference of 5.5% between GM and WM, T_1ρs_ components for GM and WM were similar. It may be possible that the difference between the GM and WM estimates is reduced due magic angle effects owing to fiber orientation^21^.

As demonstrated by Redfield^22^, in the presence of a spin lock RF field, the spins will interact with different internal Hamiltonians such as chemical shift, J-coupling, chemical exchange, and dipole-dipole interactions. In general, these interactions may be classified into three main categories (a) scalar coupling (b) dipole-dipole interaction and (c) chemical exchange processes. Depending upon the tissue environment, several mechanisms may contribute in different proportions to the T1ρ relaxation. This contribution, R1ρ (=1/ T1ρ) at a spin-lock frequency f_1_ can be expressed as:

1 $${{\rm{R}}}_{1{\rm{\rho }}}({{\rm{f}}}_{1})={{\rm{R}}}_{1{\rm{\rho }}}^{{\rm{FD}}}({{\rm{f}}}_{1})+{{\rm{R}}}_{1{\rm{\rho }}}^{{\rm{Diff}}}({{\rm{f}}}_{1})+{{\rm{R}}}_{1{\rm{\rho }}}^{{\rm{Ex}}}({{\rm{f}}}_{1})+{{\rm{R}}}_{1{\rm{\rho }}}^{{\rm{RDI}}}({{\rm{f}}}_{1})$$

where ${{\rm{R}}}_{1{\rm{\rho }}}^{{\rm{FD}}}$ is the dipolar relaxation due to molecular rotational processes (fluctuating dipolar fields), ${{\rm{R}}}_{1{\rm{\rho }}}^{{\rm{Diff}}}\,$is the diffusion contribution, ${{\rm{R}}}_{1{\rm{\rho }}}^{{\rm{Ex}}}$ is related to the exchange processes associated with water protons and other exchangeable protons on macromolecules, and ${{\rm{R}}}_{1{\rm{\rho }}}^{{\rm{RDI}}}$ is the contribution due to residual static dipolar coupling. In the presence of a spin lock field the contributions from the fluctuating dipolar fields and diffusion become negligible and the main contributions are predominantly from processes that occur with correlation times in the range of the applied spin lock frequency i.e. from the exchange and the residual dipolar interactions. At intermediate to high exchange rates, the R1ρ may be dominated by increasing contribution from chemical exchange^23,24^. Moreover, the variation of R1ρ with spin-locking power (R1ρ dispersion) can be used to estimate the exchange rate of exchanging species^23,24^. From our simulations we note that the R1ρs component contribution increases with chemical exchange and decreases with increasing concentrations at different spin-lock powers. In the brain, chemical exchange mediated by hydroxyl (−OH), amine (−NH_2_), and amide (−NH) groups with bulk water may contribute to the observed short component (T_1ρs_). In our study, we attribute the short T1ρ component to the weighted average of different chemical exchange sites, residual dipolar interactions, restricted water protons within myelin sheets and axons, with bulk water. From numerical simulations, the long R1ρ component is unaffected by fast exchange, and R1ρl linearly increases with concentrations of exchanging and non-exchanging pools but with different slopes. The long T1ρ component is attributed to free or loosely bound intra and extra-cellular axonal water. Depending on the dominant exchanging rates and their concentration fractions, the observed MRI signal would result in multi-exponential decays.

The chief constituent of egg whites is water (90%) with smaller components consisting of a variety of proteins^25^. The predominant relaxation fraction of egg white is a slow relaxing component, most likely from the water component, while the smaller fraction originating from the protein content. Egg yolk on the other hand consists of water (50%), lipids (34%) and proteins (16%)^25^. In our study, a major fast relaxing component and a minor slow relaxing component were identified. Cooking the egg from raw to soft-boiled to hard-boiled did not significantly change relaxation fractions, but they significant reduced the relaxation times for the short and long components for the egg white and egg yolk. Mitsouras *et al*. performed MCR T_2_ studies on the egg and identified non-monoexponential decays in different parts of the egg^17^. In the egg white they identified similar relaxation fractions but the relaxation times varied from our estimates. In the egg yolk they identified tri-exponential decays including a short and a long component. To isolate a third intermediate lipid component they used a fat-saturation pulse. In our study, we used binomial RF pulses to excite water protons only without touching the signal from fat. The results reported closely matched our study with the short component, but with much slower relaxation times and fractions as compared to our estimates. This may be attributed to sensitivity differences between T1ρ and T_2_ relaxation mechanisms.

We acknowledge that this study has the following limitations: The number of human subjects used was low and homogenous in age group with a range of 23 to 33 years. Although this study with healthy human volunteers did not have motion related image artifacts that may affect T1ρ quantification, the longer acquisition time can result in bulk subject motion in patient populations which can be a potential source of error in T1ρ estimates. One possible source of error in the T1ρl values is that the longest TSL (65ms *in vivo*/75ms for eggs) does not reach the longest estimated T1ρl values (upto 82ms *in vivo*/300ms for eggs). One approach to reduce the acquisition time could be to reduce the number of TSLs to the minimum required for accurate T1ρ quantification. But the redundancy in our study offers an advantage of affording the luxury of discarding a particular TSL acquisition where data corruption from movement occurred. Another approach to reduce data acquisition time would be implementing a fast parameter mapping sequence^26^. Using adiabatic T1ρ preparation, it has been demonstrated that it results in increased sensitivity to chemical exchange, and insensitivity to RF field inhomogeneities^27,28^. This would result in increased accuracy of T1ρ estimates, with increased SAR penalties and reduced specificity resulting from a broad frequency range.

In conclusion, in this study we demonstrated, using T1ρ imaging, the identification of bi-exponential decays in the brain. The short and long T1ρ values and their fractions for the whole brain can be characterized by bi-component T1ρ analysis, which is superior to single-component analysis with reduced SSE during fitting and greater information on both macromolecular bound and free water components.

## Methods

### *In Vivo* Brain Experiments

This prospective study was approved by New York University Langone Medical Center’s institutional review board (IRB) and was HIPAA (Health Insurance Portability and Accountability Act) compliant. Seven healthy volunteers (2 male, 5 female, mean age = 27 ± 4 years) with no history of neurological disease, psychiatric disease and prior head trauma were recruited following informed consent. Whole brain 3D T1ρ images were acquired with the following parameters: spatial resolution = 0.9 × 0.9 × 2 mm^3^, TR = 1500ms, TE = 4 ms, flip angle = 8°, matrix size = 256 × 256, 64 slices, field of view (FOV) = 240 mm^2^, slice thickness = 2 mm, spin lock frequency = 500 Hz, 10 TSL values = [2, 4, 6, 10, 15, 25, 35, 45, 55, 65 ms], receiver bandwidth = 515 Hz/pixel. Acceleration factor using GRAPPA = 2 was used to reduce total imaging time to ~19 min.

*In vivo* B_0_ maps were obtained for multiple slices for 1 volunteer using gradient echo phase images at two different echo times^29^. Imaging parameters were: FOV = 240 × 240 mm^2^, matrix size = 80 × 80, TR=582 ms, TEs = 4.92 ms, 7.38 ms, FA = 60°. The phase was corrected for phase wraps, and frequency offset (ΔB_0_) was calculated on a pixel by pixel basis as the accumulated phase between the two echoes. *In-vivo* B_1_ maps were obtained for multiple slices with a vendor provided rapid B_1_ mapping technique^30^. Imaging parameters included: FOV = 256 × 256 mm^2^, matrix size = 64 × 64, TR = 2000 ms, TE = 1.83ms, FA = 90°. The ΔB_1_ maps were calculated as the variation from the nominal flip angle.

### Avian Egg Experiments

As a phantom that mimics multi-component decays in living tissue, we took inspiration from a recent report^17^ and used unfertilized avian eggs to test the ability of T1ρ imaging to detect bi-exponential decays. Similar to the procedure detailed previously^17^, HB, SB and raw uncooked (n = 2) chicken eggs were used. HB and SB eggs were boiled in water for 15 and 5 minutes respectively. The eggs were then kept at room temperature for 24 hours before scanning.

T1ρ weighted images were acquired with 13 TSL intervals at 2, 4, 6, 8, 10, 12, 15, 25, 35, 45, 55, 65, 75 ms. The acquisition parameters were as follows: TR  = 1500 ms, TE = 4 ms, flip angle = 8°, matrix size = 256 × 256, 32 slices, field of view (FOV) = 200 mm^2^, and slice thickness = 2 mm, spin lock frequency = 500 Hz. The images were fully sampled, and took a total acquisition time of ~50 minutes. Regions of interest (ROIs) were drawn around the egg whites and yolks.

### MRI sequence and hardware

**Figure 6:**
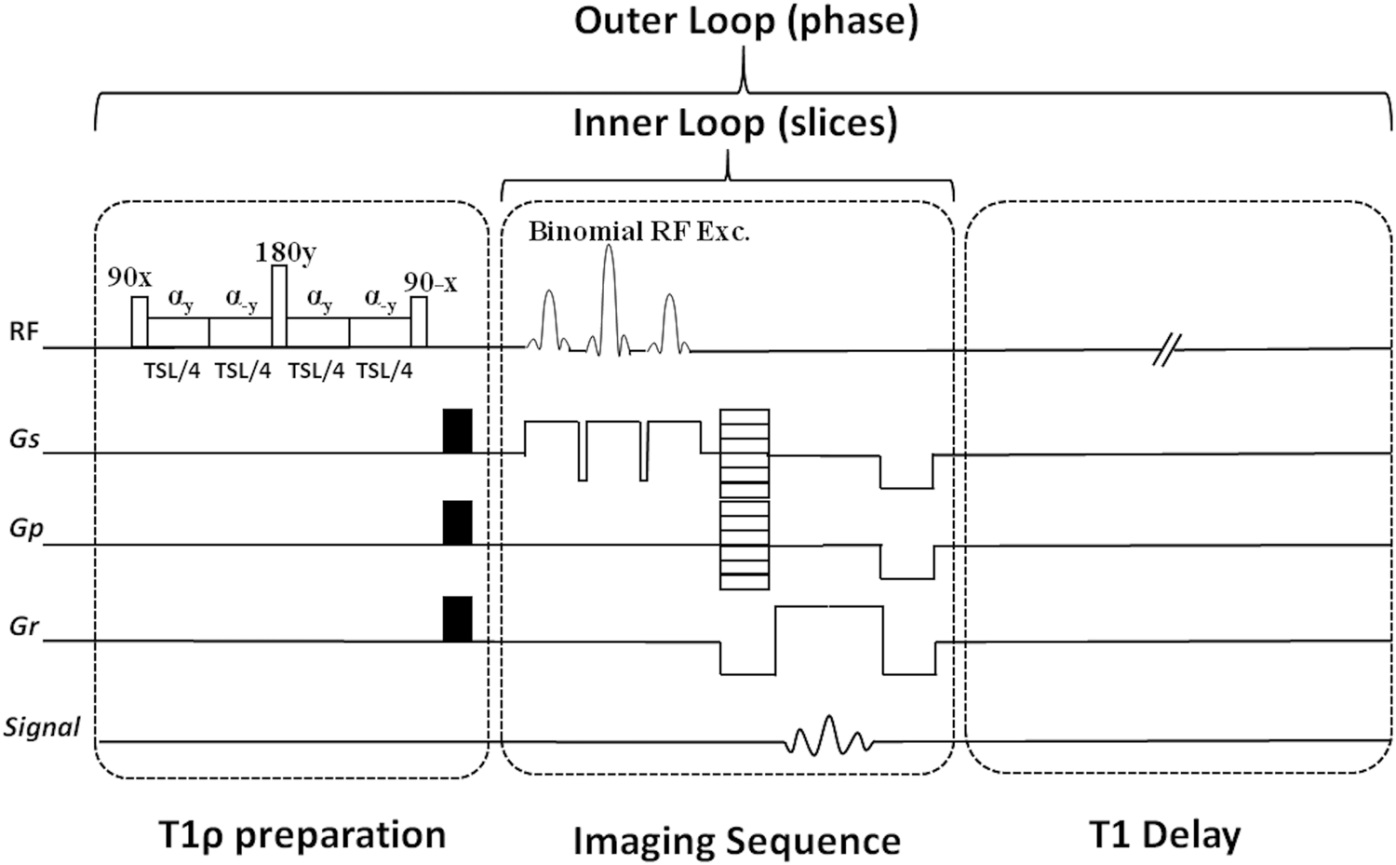
The imaging sequence timing diagram consists of T_1ρ_ preparation, 3D turbo-Flash readout, and T1 recovery delay. To compensate the effect of B_1_ inhomogeneities, the spin-lock pulse (SL) was divided into four segments with alternative phase. A 180° refocusing pulse was applied between two pairs for B_0_ inhomogeneity compensation. One phase line from all slices was acquired after applying the preparation module (inner loop). After T_1_ restoration delay the preparation module was applied again to acquire another phase line (outer loop).

All imaging experiments were performed on a 3 T clinical scanner (Prisma, Siemens AG Healthcare, Erlangen, Germany). The imaging pulse sequence is shown in Fig. 6A 3D Cartesian turbo fast low-angle shot (turboFLASH) sequence with a customized T1ρ preparation module was used to enable T1ρ imaging with varying spin-lock durations. The T1ρ preparation used a paired self-compensated spin-lock pulse to minimize B_0_ and B_1_ inhomogeneities. A binomial RF pulse was employed that uses a series of composite pulses that excites the water peak only (the fat peak remains untouched). To minimize the effects of B_0_ variations, a 180° refocusing pulse is inserted midway between the 90° pulses. To achieve B_1_ insensitivity, two self-compensated pulses of opposite phase (+y/−y), each of duration TSL/4 is applied along the y-axis. This ensures that the magnetization returns to the starting point before the 180° refocusing pulse is applied. This technique was reported earlier^31^ by another research group. For all experiments a constant spin lock frequency of 500 Hz was used. Following data acquisition, a T1 restoration delay is added for each TR. For the egg experiments a 15 channel Tx/Rx knee coil (Quality Electrodynamics Inc., Mayfield, OH, USA) was used and for the *in vivo* brain experiments a vendor provided 16 channel receive only birdcage head coil (Siemens AG Healthcare, Erlangen, Germany) was used.

### Data Analysis and Statistics

The mono-exponential T1ρ decay obtained by fitting the signal at different spin lock times for each pixel may be calculated as:

2 $$S=A\cdot {e}^{-TSL/{T}_{1\rho m}}+{A}_{0}$$

where S is signal intensity, A is the amplitude, T_1ρm_ is the mono-exponential T1ρ decay, and TSL are the spin lock times, A_0_ is the average noise level. The bi-exponential signal decay for each pixel may be calculated by fitting the data by modeling as components of two separate relaxation components as follows

3 $$S={A}_{s}\cdot {e}^{-TSL/{T}_{1s}}+{A}_{l}\cdot {e}^{-TSL/{T}_{1l}}+{A}_{0}$$

where S is the signal generated, A_s_ and A_l_ are the fractions of the signal that is contributed by the short and long components respectively, and expressed as a percentage given by a_s_ = A_s_/(A_s _+ A_l_)x100, and a_l_ = A_l_/(A_s_ + A_l_)x100. T1ρs and T1ρl are the relaxation times of the short and long T1ρ components and TSL is the duration of the spin lock.

For the egg experiments, signal decays in each ROI were fitted on a pixel-by-pixel basis with mono- and bi-exponential decays using custom scripts written in MATLAB (The MathWorks Inc., Natick, MA, USA). Hand-drawn ROI’s for the HB, SB and raw egg whites, and egg yolks were made and images from TSL = 2 ms were used to create masks for the relaxation calculations. For the human brain experiments, FMRIB software library (FSL) software was used to de-skull the images, and for segmentation into GM and WM. Binary 3D masks were made for GM, WM, GM+WM, and were used for relaxation calculations. T1ρ mapping was done by fitting the data for each pixel to the mono-exponential signal model (equation 2), and the bi-exponential signal model (equation 3). For the bi-exponential fitting, if the condition 4 × T_s_ <T_l_ is not satisfied they were excluded from the bi-exponential maps^32^.

During the fitting process, the adjusted R^2^ was computed. For both mono- and bi-exponential fits, the sum of squared errors (SSE) was used as a measure for the goodness of fit. A significant P value suggested that bi-exponential fit is better than the mono-exponential fit, with the level of significance, P <0.05, considered to be statistically significant.

### Bloch McConnell Simulations

To evaluate the effect of chemical exchange using a multi-pool model, and understand possible sources of the short and long T1ρ components, Bloch-McConnell (BM) simulations^33,34^ were employed using a 3 pool model as shown in Fig. 5(a). The model consists of a water pool (pool A), an exchanging pool (pool B) and a non-exchanging pool (pool C). There is chemical exchange between pool A and B, but minimal exchange between pools A and C, and negligible exchange between pools B and C. Data obtained from the numerical BM simulation was used to fit for the short and long T1ρ components. The spin lock frequency was varied (SL = 250, 500, 750 Hz) with an assumed spin lock duration of 55ms, exchange rates between pools B and A was varied (*k*_BA_ = 0.5, 1, 2, 3, 4, 5 KHz), the fractional population was varied (*f*_B_ = 0.005, 0.01, 0.015, 0.02, 0.025). The fractional population in non-exchanging pool varied as *f*_C_ = 0.03, 0.06, 0.09, 0.12, 0.17. The default values for the simulations used for pool B: exchange rate, *k*_BA_ = 2000 Hz, concentration, *f*_B_ = 0.015%, chemical shift of 1.0 ppm, longitudinal and transverse relaxation time = 1.5 s and 15 ms, and for pool C: *k*_CA_ = 25 Hz, *f*_C_ = 0.09%, chemical shift of 0 ppm, longitudinal and transverse relaxation times = 1.5 s and 15 μs, respectively.

### Data Availability

The datasets generated during and/or analysed during the current study are available from the corresponding author on reasonable request.
